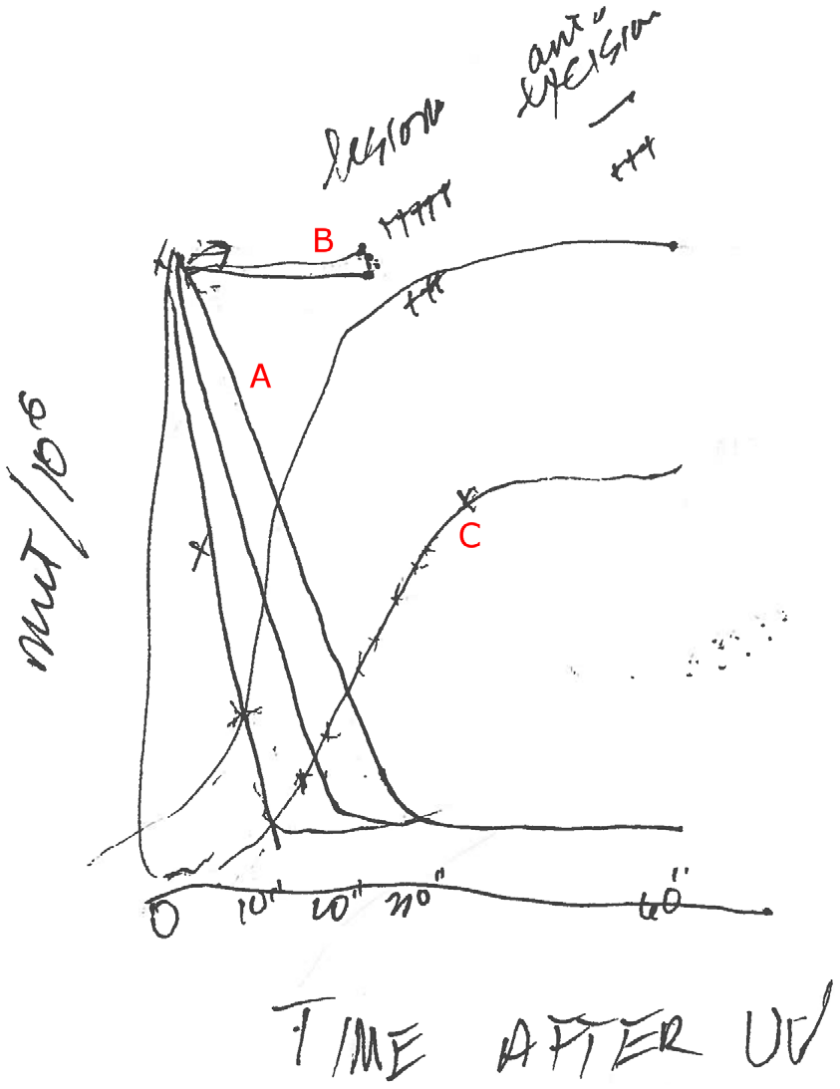# It Was Heaven: An Interview with Evelyn Witkin

**DOI:** 10.1371/journal.pgen.1003009

**Published:** 2012-10-11

**Authors:** Jane Gitschier

**Affiliations:** Department of Medicine and Pediatrics, University of California San Francisco, San Francisco, California, United States of America

Evelyn Witkin came of age at the dawn of bacterial genetics. In 1941 and with World War II looming, she began her PhD studies at Columbia University, and soon, with her very first experiment, she serendipitously cracked open a new field of research by discovering bacteria resistant to ultraviolet (UV) light.

Witkin's journey from UV resistance to DNA mutagenesis and repair spanned 50 years, taking her from Columbia University to Cold Spring Harbor Laboratory, and later from Downstate Medical Center (part of the State University of New York) to Rutgers University, from which she retired in 1991. Her original observations of filamentation associated with UV damage in *E. coli* and its suppression in a UV-resistant mutant revealed the phenomenon of cell division checkpoint and were early harbingers of the cell's SOS response to DNA damage. In the 1950s, Witkin discovered that the nature of the culture medium on which bacteria are grown post-UV irradiation strongly influences the frequency of mutations that arise: high on media that promote active protein synthesis, but far lower on media that support protein synthesis only after a significant delay. Witkin studied the kinetics of this phenomenon, soon referred to as “mutation frequency decline” (Mfd), and showed that if protein synthesis is delayed or inhibited, genetic damage is corrected rapidly by an enzymatic repair mechanism (later proven to be excision repair of pyrimidine dimers), whereas if protein synthesis is enabled, abundant mutations are generated as products of error-prone repair, part of the manifold SOS responses.

What is remarkable is that many of these early observations and hypotheses originated before it was even appreciated that DNA was the genetic material or that UV damaged DNA directly. Like other pioneering geneticists of that period—Barbara McClintock with her maize or Mary Lyon with her mice—Witkin brought tenacity and powerful skills of observation to develop a deep understanding of a fundamental problem in biology.

**Image 1:**
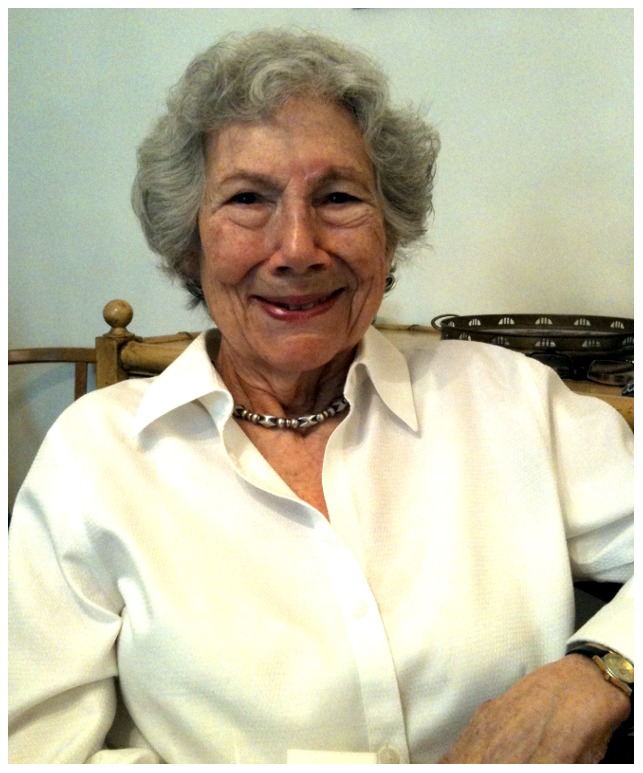
Evelyn Witkin.

Upon interview, I discovered that Witkin (Image 1) also infused a special attribute into every aspect of her life: joy. On a grey morning in early May, I made my way to her home at the end of a cobbled cul-de-sac, a half block from the Princeton campus, and she welcomed me with a strong cup of coffee and a youthful effervescence that belies her age. Though no longer practicing science, she has an active life involving historical research on Charles Darwin and Robert Browning, visits to her family in California, reading about cosmology with the Princeton Research Forum's science book group, and, of course, graciously entertaining visitors like me.

**Gitschier:** What prompted you to retire?

**Witkin:** At the time, it was the law that you had to retire at 70. I was 70 in 1991, so do the arithmetic.

**Gitschier:** Wow, you're getting up there! You look fantastic!

**Witkin:** Thank you. Anyway, I could have stayed on year-by-year at Rutgers, but I decided not to because I felt that the field was somehow getting away from me. And I didn't want to push my luck with grants, because the way I like to work was not the way things were going at the time. I did most of my experiments myself with my own two hands; I had a small group, just two or three graduate students. I had no post-docs. And I felt that things had changed and one needed to have larger groups with various types of expertise represented. I had the same grant essentially since 1956! And I felt that I would have a hard time asking for money at that point.

**Gitschier:** So you worked in the lab all through your sixties?

**Witkin:** Oh gosh, yes. I mean I started in the '40s. I was a graduate student from 1941 to 1947 when I got my PhD. And that was an interesting time to be starting. Especially at Cold Spring Harbor!

**Gitschier:** I'll bet. I'm very curious about your upbringing and how you met your husband. What was his name?

**Witkin:** Herman A. Witkin. “Hy” was his nickname. I met him through my sister. She was a graduate student in psychology at New York University when I was an undergraduate [there]. As a matter of fact, I was a 16-year-old freshman when I met him.

**Gitschier:** Did you grow up in Manhattan?

**Witkin:** No. I was born in Manhattan, grew up there my first 9 years and then my mother re-married. My father had died when I was three. And when my mother remarried, we moved to Queens. My stepfather had just built a fabulously elegant “drug store”, but it was a whole lot more than that. It had a food service, which was almost like a very good restaurant, and my mother supervised the food service. It was in the building that we moved into, actually—a big apartment. It was the most outlying part of Forest Hills; everything else was field beyond that point. You'd never know it now.

And I commuted to high school, before there was a subway to Forest Hills on Long Island. I had to take the Long Island Railroad at 6:59 every morning.

**Gitschier:** You still remember the time!

**Witkin:** Yes, I remember it was mostly dark at that hour. It was not a very nice trip. The train took only about 20 minutes, and then I had to walk through a long dark tunnel and then take the subway to Union Square, and then walk to Washington Irving High School. I thought nothing of it, of course, at the time. I was 12 when I started high school. And it was a wonderful place to be.

My stepfather was very firmly convinced that Washington Irving, which at that time was a public all-girls school, was the best high school in the area and that we should go there. So both my sister and I did, although it was a nasty commute.

But I am so glad that I went there. It was the best educational experience I think I have ever had. It changed my whole way of thinking about everything! I just loved that place. And I was so turned on about learning about everything. The excitement of that was tremendous. And the interesting thing is that I have a lot to do with it now.

**Gitschier:** Really?

**Witkin:** I'm a Robert Browning enthusiast. And the New York Browning Society meets at the National Arts Club at Gramercy Square in Manhattan. And one or two blocks from there is what was Washington Irving High School. The building is still there, and it now houses four high schools. I graduated in 1937. You're not going to believe these dates, they go back!

I hadn't set foot in that building since I graduated. And then, the president of the Arts Club was very eager to do some neighborhood outreach. He thought we ought to bring the students at Washington Irving High School into our activities. So I was elected to make contact because I was an alumna.

Every year we have a poetry contest for high school students, and the winners read their poems at the annual luncheon. And Washington Irving won one of the years, and that was lovely.

**Gitschier:** Why did you get to high school at such a young age?

**Witkin:** Well, in the days when I was in elementary school, they used to skip people right and left. And my sister and I both skipped every other half of the year—there was a 1A and a 1B the first year and a 2A and a 2B. We skipped all the Bs for the first few years! That was too young—to start college, at 16. I was so unready for it! Not academically, but socially. Coming from an all-girls high school, I had never talked to a male! I grew up in a household of females, mostly.

**Gitschier:** OK, now back to NYU and the question of how you met Hy.

**Witkin:** He was part of my sister's group of graduate students that moved together a lot. And I was a freshman and he was almost 5 years older than I. These were the days of the Spanish War, 1937 to '38. And I was already very political, very left. And I was passionately in support of the loyalists in Spain. And there was a fair held in a little Greenwich Village mews near Washington Square to raise money for the loyalists, and my sister and her group were going and she asked me to come, too. That is where I met Hy.

I thought of him as another generation! He was older than my sister, so I never thought of him in that [romantic] way. But he insisted later that it hadn't taken more than one look for him to decide I was it for him! It took us five years before we married. I was just 22.

**Gitschier:** And that year would have been …

**Witkin:** '43. He was 26 when we married, so he was already established with a faculty job in psychology in Brooklyn College. And I was 2 years into my graduate work at Columbia. And for one wonderful summer we lived in Washington Square in a hotel while we looked for a place to live. We found this wonderful studio apartment on 2 Horatio Street in the Village.

At the end of that first year, Hy went to work with the School of Aviation Medicine in Texas. This was of course during the war, but he was not in the army yet. His research was on space orientation, and the Air Force was very interested because he was working on how people determined what is upright. When a pilot is flying upside down it is important for him to figure that out! So they wanted him to do his research there, and that's when I went to Cold Spring Harbor.

**Gitschier:** Now, initially you had planned to work with [Theodosius] Dobzhansky at Columbia. Tell me about that.

**Witkin:** I had this very radical boyfriend when I was 18. He was a Harvard student—my first real boyfriend. And he was even more radical than I was, and I was pretty hot!

He had come across English translations of some articles by Lysenko. And I had not yet gotten very deeply interested in genetics. And we read these articles together, and I thought it sounded very interesting, that you could manipulate the environment and change heredity in any desired direction. And I thought, hmm, maybe that is something I can work on when I get to the point of choosing a research topic. I'd like to see if he is right.

So that is what hooked me on genetics! And when I got to Columbia, the reason I wanted to work with Dobzhansky was not because he was a great geneticist—I didn't know that—I didn't know anything! But he was *Russian*, and he would be able to read Lysenko's papers in the original [language]. So he's the one I asked, “Could I be your student?” He had never had a female graduate student, and he thought that was kind of intriguing, and he said, “Sure.”

So then I started graduate school at Columbia, and it took me about 3 months to be cured of Lysenko, because I took a really hard, serious genetics course, and I went more deeply into it and realized that he was probably a fraud. So that was that.

**Gitschier:** Let's talk about the transition to Cold Spring Harbor.

**Witkin:** What happened was that I was taking a class with Dobzhansky, and he gave me a pre-print of the Luria and Delbruck paper, which of course proved that bacteria have genes like everybody else, and I was supposed to report on that in a class. And I did it jumping up and down with excitement, because I thought, oh boy, this is opening up a whole new way of doing genetics, where they multiply every 20 minutes and a billion of them fit into one little test tube. So he sensed my interest in this and said, “If you think this stuff is so exciting, why don't you go out to Cold Spring Harbor this summer? Luria and Delbruck will be there.” He was a friend of [Milislav] Demerec, the director of the Department of Genetics of the Carnegie Institute of Washington, which was also located at Cold Spring Harbor at the time, and he spent his summers there, too.

**Gitschier:** Oh? Did he work on flies during the summer?

**Witkin:** Yeah. There was a group that worked on flies in the summer. There were very few year-round people then.

**Gitschier:** You know, it just seems like such a lovely experience!

**Witkin:** Oh, it was heaven. So, never having had a course in microbiology, not knowing a thing about sterile technique, nothing about bacteria, I went to Cold Spring Harbor for the summer. He [Demerec] sat me down at a UV lamp and said, “Go, induce mutations.” And I had no idea where to begin. Somebody helped me out, so I learned how to start a culture and do all that stuff.

**Gitschier:** Now, let's just back up one second. I'm curious about the history of UV irradiation. What was known at that time, and why did Demerec even have a UV General Electric germicidal lamp?

**Witkin:** Well, I think it was around 1928 when [Hermann] Muller discovered that X-rays could induce mutations.

**Gitschier:** Yes, and that was in *Drosophila*.

**Witkin:** Yes. At the same time Muller was doing that with *Drosophila*, another member of the Morgan group, which had scattered, had been looking at mutagenesis with ultraviolet light. [Edgar Altenburg, a colleague of Muller's at Rice University, was the first to induce mutations with UV, also in *Drosophila*.]

By the '40s, certainly Demerec was not the only one who was trying to induce mutations with ultraviolet light. He was still working with *Drosophila* then, but he had just turned to bacterial genetics. He was one of the only ones of the classical group who had switched to bacteria.

**Gitschier:** So he was really perfect for you.

**Witkin:** He was. He had a set up, and he was doing wartime things for the government. Antibiotics had just been discovered, and he was looking to get mutations resistant to antibiotics.

**Gitschier:** To figure out …

**Witkin:** How they kill.

**Gitschier:** Was that because people were already starting to have resistance?

**Witkin:** No, they weren't even thinking about it. The geneticists were the first to warn that you have to use more than one [antibiotic] at a time if you want to prevent resistance. Certainly nobody was thinking that in the medical profession. That's why he had a UV lamp. He was doing war work. And he was trying to get mutants that made more penicillin, things like that. He was using the UV as a potential mutagen because it had been established that it was mutagenic.

Hollaender and Emmons showed in 1941—this was before DNA was at all established [as the genetic material]—that the absorption spectrum for UV light matches the action spectrum for inducing mutations in fungi. And they superimposed the peak of effective absorption with the peak of mutagenic activity. [Indeed, the relationship between the bactericidal effects of UV and its absorbance by nucleic acid was first pointed out in 1928 by Frederick Gates, who commented on the “significance of these substances as essential elements in growth and reproduction.”]

**Gitschier:** Yeah, the peak of absorption by nucleic acids. Which should have told everybody right then …

**Witkin:** Exactly! Because I was very excited when I saw that. I think it is a very important early hint. More than a hint!

**Gitschier:** Historically, it is a very important paper.

**Witkin:** And you know, they wriggled out of it with all kinds of excuses.

**Gitschier:** In the paper?

**Witkin:** Yes. In the last paragraph, they proposed that the nucleic acid could be the absorbent, but that the energy was then transferred to protein!

But you know, they were so convinced—Demerec, Dobzhansky, this whole group in Cold Spring Harbor, all the geneticists there, including myself, were absolutely convinced that the genetic material had to be a protein.

**Gitschier:** What about Luria and Delbruck?

**Witkin:** We *all* thought that. Because the biochemists told us that DNA was a single chain of repeating tetranucleotides; Delbruck said, “That's a stupid molecule. It couldn't possibly be the gene.” So we believed the biochemists. So they had to wriggle out of this evidence.

But that's an example of how people were looking for reasons not to believe this result. Not to make the obvious conclusion. That was a very solidly felt prejudice: that it had to be a protein.

And even in 1944 when we read the classic paper by Avery, MacLeod, and McCarty, nobody believed it in Cold Spring Harbor! They wriggled out of that one, too. It was a lesson for the need for a little skepticism at all points.

**Gitschier:** And it's interesting because we do the same thing over and over again.

**Witkin:** We always do. What doesn't fit is often what is getting at something exciting! Quite often.

**Gitschier:** Sometimes it is hard to figure that out, though, at the beginning.

**Witkin:** But I think the best scientists have a nose for that—deciding what to follow up when something doesn't fit. And you can be wrong, no matter how good you are.

**Gitschier:** So, that summer of '44, when you were there at Cold Spring Harbor, had your husband already moved to Texas?

**Witkin:** Yes. That's why I could move to Cold Spring Harbor.

**Gitschier:** So rather than *inducing* mutants, as Demerec had instructed, you discovered radiation-*resistant* mutants.

**Witkin:** Yes, and that became my PhD problem very quickly. And I worked on it over the summer and thought I'd go back to Columbia and continue to work on it. But Columbia was definitely *not* a microbial department. Everything was so slow because of the logistics of trying to get things sterilized and do things. So Dobzhansky—again, he was really so helpful to me—recognized this, and he said, “You know, it would be very good if you could go back to Cold Spring Harbor and stay there until you could finish your project.” And by the end of '44 I was finished with my courses, so I could go out there and stay and pursue my research.

**Gitschier:** And your husband went into the army. When was that?

**Witkin:** It was late '44 or early '45. There was no break in my freedom to go to Cold Spring Harbor. When he came out of the army, he came to Cold Spring Harbor and commuted to Brooklyn College. We didn't realize it at the time, but he was going to be doing that for 10 years!

**Gitschier:** Now, back to this experiment. You basically turned the UV up too much, killed everything, but got these four resistant colonies. You worked with an *E. coli* strain called B, which was the wild-type strain that Demerec had in the lab and which he was using. And it was of a completely separate origin from the K12 strain.

**Witkin:** Right. If Cold Spring Harbor had been using K12, I wouldn't have discovered radiation resistance. It was because of a peculiarity—a genetic mutation—in the B strain that made that possible. I think I'd better start at another point.

**Gitschier:** Sure.

**Witkin:** Luria suggested, “Take a look at these bugs under the microscope.” And so, I irradiated the B bacteria on a Petri dish …

**Gitschier:** … at a dose that was far lower than the dose you used to isolate resistant mutations, as I recall.

**Witkin:** Yes, a very low dose, and looked at them on the surface of the plate and found that they formed long, narrow filaments. And *that* was the reason that they were sensitive to UV.

I was able to show in my thesis that their sensitivity was due to this inhibition of cell division. We could stain something that looked like a nucleus—of course, the DNA—and could see that these little dark spots are populating this filament, but we're not getting the forming of septa. And within a matter of 3 hours, they reach a length of about 50 times normal length, and then they die, and this is why they are sensitive to UV. And this is 100% of the cells.

**Gitschier:** So, if you had exposed K12 to the same dose of UV, they are going to keep dividing, and hence they are relatively more resistant?

**Witkin:** I did that later. What I found was when you irradiate [K12] with the same dose that you use on the B strain that makes them filament, they elongate slightly to about two or three times normal length and then they start splitting off new cells. They all survive this very low dose.

Later on we learned how this works, and maybe I'd better just jump to that. What we really found out was that when the DNA is damaged, there is an inhibition of cell division. And when repair is complete, that inhibition of cell division is eliminated, and septation resumes. And that is in K12.

**Gitschier:** Does this delay have an official name?

**Witkin:** Checkpoint. We didn't call it that back then, but in a way, this is the first demonstration of a cell checkpoint. I was fascinated with the microscopic part of this. I just did endless experiments to record what they did at various doses and have drawings of those things. We didn't really know what the genes were yet, but we knew it [the mutation in the B strain] involved an inhibition of septation.

Later on, it was found that the mutated gene in the UV-resistant mutant strain [named “B/r”] was one of the SOS genes. It was interesting that my first lucky strike turned out to be one of the SOS genes; SOS was not discovered until ‘72, and this was ‘43. It's called *sfi* [suppressor of filamentation].

The Sfi protein is a cell division inhibitor that is induced as a part of the SOS response. And it is induced in K12 and it is induced in B. And it strictly forbids septation. The B/r strain has the normal SOS response *except* that it doesn't make the cell division inhibitor. So, it doesn't elongate, the way K12 does, even a little bit. It *never* elongates!

Then, there is a protease that rapidly degrades the cell-division inhibitor, once repair is complete, and the B strain has a defect in this protease gene!

**Gitschier:** And what is this protease called?

**Witkin:** Lon. So the B strain is defective in the Lon protease that normally degrades the cell division inhibitor Sfi. This was all worked out later by Susan Gottesman at NIH. And therefore, the cell division inhibitor persists and continues to inhibit septation long after repair is completed and SOS is turned off. So, if you give a low dose, you can see this filamentation going on for 3 hours before they die. There is a limit as to how long they can go on without dividing. They just die.

**Gitschier:** So, they are not dying because the DNA is damaged per se …

**Witkin:** No!

**Gitschier:** Oh, neat! It's nice to go back and put the puzzle together. What would you say was the next big thing scientifically for you?

**Witkin:** I guess Mfd, in 1952 or '3. Which is a hard thing to explain to anybody.

**Gitschier:** Do you feel like you have the courage to do that?

**Witkin:** It was a very difficult puzzle and the value of it was in the use of it as a way to study mutagenesis, even without understanding what was happening in this phenomenon. It provided us with a system of quantifying induced mutations and of seeing repair before our eyes, almost.

*[The intrepid reader is referred to the addendum for a historical account and explanation of Mfd.]*

**Gitschier:** In fact, you were actually able to predict an enzymatic repair mechanism on the basis of Mfd.

**Witkin:** Yes. I predicted excision repair before [Richard] Setlow found it. I called it “dark repair” to contrast with photoreactivation [which had been discovered in 1946 independently by Albert Kelner and Renato Dulbecco]. In fact, there were two things I learned from Mfd …

**Gitschier:** … the other being the idea of error-prone repair. So let's move ahead to the SOS response. In 1967, you had a *PNAS* paper where you talk about the relationship between prophage induction and filamentation in response to UV.

**Witkin:** Yeah, that was an early SOS hint. Two of my papers in '67 hinted to SOS. The other was the Brookhaven Symposium paper. I published in obscure places. The reason was that I never wrote unless I had to give a talk somewhere. At Cold Spring Harbor, there was no pressure to publish. No pressure! So when I got invited to give a talk, I would write it up.

Anyway, the Brookhaven paper and the *PNAS* paper are both hinting at the SOS system. The one in *PNAS* is doing it by showing that induction of filamentation parallels the induction of prophage, and that suggests they are under coordinate regulation. And then I also say that maybe there are other bacterial genes, not just phage genes, that are similarly induced. The Brookhaven paper specifically says that the way mutagenesis seems to happen is that when a lesion is not excised and the replication complex gets to it, there is a mechanism that inserts something so it can survive, and the chances are high that there is going to be an error, a wrong insertion. The name of that paper was “Mutation-proof and mutation-prone modes of survival in *E. coli*,” and this was proposing error-prone repair as the mechanism for UV mutagenesis.

That paper does not propose induction of it. But it does propose error-prone repair. The other is proposing induction. That's why I always cite these two '67 papers in the history of SOS.

**Gitschier:** Right, they are complementary.

**Witkin:** You see, [Miroslav] Radman was the one who proposed the induction of the SOS mutagenesis. I didn't know it was induced till he proposed that.

Radman was making a strong case for what he called “SOS replication,” the induction of an error-prone repair. But he didn't conceive of it as being a broader complex and I did, as I had already thought of it that way. I thought, well, I'd better look at the literature and see what UV effects require protein synthesis to be manifest, and I made a table of everything I could find and it was a lot of stuff, and I proposed that there was an inducible cluster. So that was a very exciting experience. By 1974, I had provided definitive experimental proof that SOS mutagenesis was inducible as a component of the SOS response. Radman was right.

We had a meeting in 1975 in Gainesville, Florida, which is where a lot of people, who had little pieces of the SOS puzzle and didn't know about the overall cluster, came together. And it was a very exciting meeting because there were only a few of us—Radman and I, Alexander Hollaender, and I think by then our French colleague [Raymond] Devoret—who knew about this cluster of commonly regulated genes. The other people who came knew about their one piece, and they didn't know about the others.

**Gitschier:** What fun for them! So after this amazing career, you had to retire.

**Witkin:** If I had a couple of million dollars to build a lab in my basement, I would have gone on. *E. coli* still has lots of secrets!

**Interpreting Mutation Frequency Decline**

**Gitschier:** OK, let's talk about your discovery of Mfd.

**Witkin:** I wanted to study the induction of mutations quantitatively by UV, and to do that, I had to have a system that would reliably let me quantify. And the system I chose was using auxotrophs, which require a growth factor, and then selecting for mutants no longer requiring the growth factor.

**Gitschier:** Which Demerec was already working on.

**Witkin:** Right. Demerec grew his UV-irradiated strains on a minimal media supplemented with a small amount of nutrient broth to allow a few divisions on the plate in order to fix the mutation. When I started my experiments, I used a strain requiring tryptophan as my standard. So I thought instead of using nutrient broth as the supplement, I'd use a small amount of tryptophan, and it would be cleaner.

But I found when I supplemented with nutrient broth, I got hundreds of mutants on the plate, and when I supplemented with an equivalent amount of just tryptophan, I got hardly any. And that's the Mfd phenomenon: the difference between what you get with a broth supplement and what you get with a supplement of only one amino acid.

And when I told Demerec about this, he said, “Yeah, we noticed that, that's why we used the broth. We just didn't get any mutants on the minimal.” So, he just brushed it off, and I thought, “Why?”

**Gitschier:** And that was how you started to systematically transfer UV-irradiated cells from media with nutrient broth to tryptophan, and vice versa.

**Witkin:** Yes. And that led to seeing the kinetics. I can draw the kinetics.

**Gitschier:** Wow! This is exciting. I'm getting a personal drawing. I'll bet you haven't drawn a curve in a while.

**Witkin:** No, I haven't. And this is zero, 10, 20, 30 minutes, and this is the curve; depending on the UV dose—the lower dose, it [the mutation frequency] comes down faster.

*[See the set of curves labeled “A”: Here, UV-irradiated cells are plated on tryptophan and transferred to nutrient broth plates at the times indicated.]*

**Gitschier:** So, you are actually watching the UV damage being repaired.

**Witkin:** Yes, we are watching the excision of the damage, not the making of the mutation. What this curve is describing is excision repair going on. When a UV lesion is excised, it cannot make a mutation. A mutation occurs when an error-prone enzyme goes past a lesion that is still in the DNA. Mfd kinetics told us that excision is relatively slow and inefficient on nutrient both, so that a lot of unrepaired lesions remain in the DNA to cause mutations. On tryptophan, excision repair is much more rapid and efficient, so that after 10 minutes, only a few UV lesions remain in the DNA to cause mutations.

**Gitschier:** So you are saying that this excision repair works super fast.

**Witkin:** Yes. Mfd is actually a special kind of excision repair, confined to the transcribed strand: transcription-coupled repair, which is very rapid. [This was later proven by Christopher Selby, Witkin, and Aziz Sancar using an *mfd* mutant Witkin had identified.] And it seems, at least in this case, that this special kind of excision repair is inhibited by active protein synthesis [in nutrient broth] but enhanced when protein synthesis is briefly repressed right after UV exposure, as is the case with just tryptophan in the in the medium.

This gave us a way of manipulating conditions that have an effect on the frequency of mutations, and that was amazing. For example, when we look at the kinetics this way, if we put in the medium a low level of a nucleic acid dye, like acriflavine or crystal violet, mutation frequency stays up; there is no Mfd.

*[See the set of flat curves labeled “B”.]*

Acroflavin is known to intercalate between the bases. The dyes stretch out the DNA and the enzymes can't latch on the way they usually do, and they can't remove the lesions.

Shortly after Setlow and Carrier discovered excision repair [in 1962], I visited Oak Ridge and I gave Setlow a list of all of the agents that inhibit Mfd. I bet him that they would also inhibit excision of pyrimidine dimers from DNA, and that's exactly what he found. I thought we were betting a chocolate soda, but Setlow sent me a preprint of his paper inscribed, “You win the scotch.” And of course I hate scotch.

So the other kinetics I wanted to show you are the kinetics of fixation [of mutations].

*[See the two sigmoid curves labeled “C”. Here, UV-irradiated cells are plated first on nutrient broth and than transferred to tryptophan plates at the times indicated.]*

Here, we want to know when is the mutation actually fixed so it can't be eliminated anymore, and we get very different kinetics. You get them off the nutrient broth plate and put them on the tryptophan medium that doesn't promote protein synthesis. Initially, there is no fixation at all, but then you'll get some mutations and that depends on how many lesions have been excised. The ones that are excised will never make a mutation. For the first 30 minutes on nutrient broth plates, most of the induced mutations remain subject to Mfd—that is, to excision repair—after transfer to tryptophan. Between 30 and 70 minutes on nutrient broth, more and more of the induced mutations have been fixed by the time of the switch to tryptophan, and after about 70 minutes or longer on nutrient broth, the switch to tryptophan can no longer reduce the number of induced mutations. We found that the kinetics of mutation fixation could be entirely superimposed on a curve showing the synthesis of DNA in the first replication after UV irradiation. So we learned that DNA replication is required for the irreversible fixation of UV-induced mutations.

**Figure:**